# Contribution of Mobile Group II Introns to *Sinorhizobium meliloti* Genome Evolution

**DOI:** 10.3389/fmicb.2018.00627

**Published:** 2018-04-04

**Authors:** Nicolás Toro, Francisco Martínez-Abarca, María D. Molina-Sánchez, Fernando M. García-Rodríguez, Rafael Nisa-Martínez

**Affiliations:** Structure, Dynamics and Function of Rhizobacterial Genomes (Grupo de Ecología Genética de la Rizosfera), Department of Soil Microbiology and Symbiotic Systems, Consejo Superior de Investigaciones Científicas, Estación Experimental del Zaidín, Granada, Spain

**Keywords:** catalytic RNAs, genome evolution, intron, insertion sequences, retroelements, rhizobia, reverse transcriptase, ribozymes

## Abstract

Mobile group II introns are ribozymes and retroelements that probably originate from bacteria. *Sinorhizobium meliloti*, the nitrogen-fixing endosymbiont of legumes of genus *Medicago*, harbors a large number of these retroelements. One of these elements, RmInt1, has been particularly successful at colonizing this multipartite genome. Many studies have improved our understanding of RmInt1 and phylogenetically related group II introns, their mobility mechanisms, spread and dynamics within *S. meliloti* and closely related species. Although RmInt1 conserves the ancient retroelement behavior, its evolutionary history suggests that this group II intron has played a role in the short- and long-term evolution of the *S. meliloti* genome. We will discuss its proposed role in genome evolution by controlling the spread and coexistence of potentially harmful mobile genetic elements, by ectopic transposition to different genetic loci as a source of early genomic variation and by generating sequence variation after a very slow degradation process, through intron remnants that may have continued to evolve, contributing to bacterial speciation.

## Introduction

Rhizobia are soil bacteria that elicit root nodules on leguminous plants (Oldroyd et al., 2011; Haag et al., 2013), in which they transform nitrogen (N_2_) from the atmosphere into ammonia. *Sinorhizobium meliloti* and *Sinorhizobium medicae* are closely related species that form nitrogen-fixing symbioses on *Medicago* plants. These two bacterial species have a large composite multipartite genome with a chromosome (∼3.65 Mb) and two large symbiotic (Sym) megaplasmids of ∼1.3 and ∼1.6 Mb in size, and some strains also have smaller accessory plasmids (Barloy-Hubler et al., 2000; Barnett et al., 2001; Capela et al., 2001; Finan et al., 2001; Galibert et al., 2001; Reeve et al., 2010). These species are found in the soil, the rhizosphere, and within root nodules. The different ecological niches colonized by these bacteria, the boundaries of gene flow between these microhabitats and plant selection have shaped the evolution of these bacterial species.

Whole-genome sequence analyses have been performed for *S. meliloti* and *S. medicae* (Bailly et al., 2011; Galardini et al., 2011, 2013; Epstein et al., 2012; Sugawara et al., 2013), and some studies have indicated that the pSymB megaplasmid plays a key role in intraspecies differentiation (Galardini et al., 2013, 2015). Whole-genome sequencing of isolates from a natural *S. meliloti* population (GR4-type) isolated from *M. sativa* (alfalfa) root nodules revealed the existence of genotypic variation underlying early genetic and ecological differentiation (Toro et al., 2016, 2017). In addition to the small numbers of single-nucleotide polymorphisms uniformly distributed over the multipartite genome, the isolates analyzed exhibited other types of genomic variation, indels of various sizes, some recombination events including the excision and acquisition of genomic islands, and the transposition of mobile elements, which seem to be the early microevolutionary forces influencing this multipartite genome. Mobile genetic elements have caused genetic variation in the three main replicons, and the variation due to one group of mobile elements, group II introns, is particularly interesting (Lambowitz and Zimmerly, 2011; Novikova and Belfort, 2017; Toro et al., 2017). These mobile introns are self-splicing RNAs and retroelements, which are highly abundant in *S. meliloti* and, to a lesser extent, in closely related species. A number of studies on the mobile group II intron RmInt1 (Martínez-Abarca et al., 1998) harbored by most *S. meliloti* strains (Muñoz et al., 2001; Fernández-López et al., 2005; Molina-Sánchez and Toro, 2015) have provided clues to the dynamics and behavior of these retroelements and their possible contribution to the short- and long-term evolutionary events shaping these bacterial genomes.

In this review, we provide a brief overview of what is presently known about group II introns, including RmInt1 in particular, in the context of their possible contribution to *S. meliloti* genome evolution.

## Main Features of Group II Introns and RmInt1

Group II introns were originally identified in the mitochondrial and chloroplast genomes of lower eukaryotes and plants (Michel et al., 1989), and subsequently identified in bacteria (Ferat and Michel, 1993), and in some archaeal species (Dai and Zimmerly, 2003; Toro, 2003).

**FIGURE 1:**
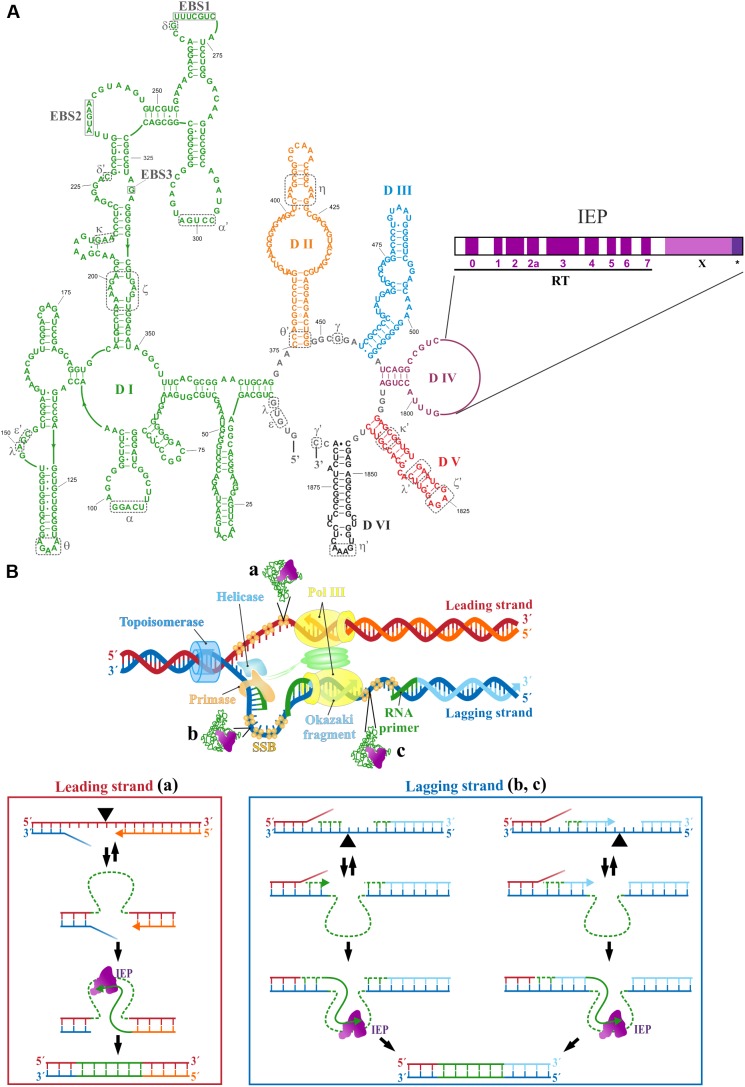
**(A)** The secondary structure of the RmInt1 ribozyme is shown. Intron RNA domains (DI–DVI) are shown in different colors. Nucleotides identify as exon binding sites 1, 2, and 3 (EBSs) in DI are boxed. Nucleotides involved in tertiary contacts are indicated by dashed gray lines and Greek letters. The intron-encoded protein (IEP) domains encoded by DIV are also shown. RT: reverse transcriptase domain with conserved RT sequence blocks (0–7); X: maturase domain; ^∗^C-terminal tail. **(B)** A schematic diagram of the replication fork with intron insertion in the template for leading strand (a), and lagging strand (b,c) is shown. The process of intron integration is represented below. Once the intron RNA reverse splices into the target DNA, the IEP retrotranscribes the RNA into cDNA, using the nascent leading strand (a), the 3′ end of the RNA primers synthesized by the primase (b), or the nascent Okazaki fragments (c) as the primer. Green dashed lines represent the RNA molecules. The black triangle indicates the intron insertion site.

Despite the fact that the nuclear genomes of eukaryotes do not contain group II introns, the nuclear spliceosome and spliceosomal introns are thought to have evolved from mobile group II introns (Koonin, 2006). Moreover, group II introns have also been identified as the probable evolutionary ancestors of non-LTR retrotransposons, telomerase, and retroviruses in eukaryotes (Lambowitz and Belfort, 2015). Group II introns have been maintained in bacteria because they home preferentially to sites outside of functional genes, within intergenic regions or in mobile genetic elements behaving predominantly as retroelements (Simon et al., 2008; Chillón et al., 2011; Nisa-Martínez et al., 2013). Typical group II introns comprises six typical stem-loop domains (DI–DVI), with an internal open reading frame (ORF) encoding an RT-maturase within DIV (**Figure 1A**). Exon-binding recognition sequences (EBS) have been identified in DI, and the most important catalytic residues are present in DV, whereas the branch point adenosine, the nucleophile responsible for initiating the splicing reaction resulting in intron lariat formation, is located in DVI. The intron-encoded protein (IEP) is a multidomain protein consisting of a reverse transcriptase (RT) domain followed by a maturase (X) domain. The RT domain resembles to the finger and palm subdomains of a polymerase, and the X domain may be equivalent to a polymerase thumb domain. Some IEPs have an endonuclease domain (EN) involved in intron mobility at their C-terminus (Belfort et al., 2002; Lambowitz and Zimmerly, 2004; Toro et al., 2007). The intron lariat (Costa et al., 2016), the RT domain without the thumb domain (Zhao and Pyle, 2016), and a full-length (Stamos et al., 2017) group II intron RT have been crystallized. The cryo-EM structure of a group II intron and its reverse transcriptase has also been reported (Qu et al., 2016).

The non-coding portion of group II intron RNAs has coevolved with their IEPs, and on the basis of conserved RNA structure, group II introns have been classified in three main classes (IIA, IIB, and IIC) (for a review see Lambowitz and Zimmerly, 2011). On the basis of their IEPs they are classified in several classes and varieties: A, B, C, D, E, F, G (*g1*), CL1 (chloroplast-like 1), CL2 (chloroplast-like 2), and ML (mitochondrion-like) (Simon et al., 2008; Toro and Martínez-Abarca, 2013).

Group II intron splicing involves two sequential transesterification reactions (de Lencastre et al., 2005; Marcia et al., 2013; Kruschel et al., 2014; Zhao and Pyle, 2017). The first reaction involves a nucleophilic attack on the 5′ splice site by the 2′-OH of a bulged adenosine residue (bulging A) located in DVI, releasing the 5′ exon and generating an intron-3′ exon branched intermediate. The second step involves a nucleophilic attack on the 3′ splice site by the free 3′-OH of the 5′-exon, yielding the ligated exons and the intron lariat RNA with a 2′-5′ phosphodiester bond. The intron binding sequences (IBS) at the 5′ and 3′ splice sites are recognized by base-pairing with the intron RNA exon-binding sequences (EBS). The EBS1–IBS1 interaction is essential for 5′ splice site recognition during the splicing reaction whereas the EBS2–IBS2 pairing is dispensable for splicing (Barrientos-Durán et al., 2011).

The mechanism of group II intron mobility was initially established (Zimmerly et al., 1995) for yeast introns (aI1 and aI2) and for the *Lactococcus lactis* Ll.ltrB intron. The mobility of these introns is mediated by a target DNA-primed reverse transcription mechanism (TPRT endonuclease-dependent) involving a ribonucleoprotein (RNP) complex containing both the intron RNA and the IEP (Lambowitz et al., 1999). Once the target sequence (homing site) has been recognized (20–35 bp) mainly by the RNA component of the RNP complex, through base pairing between the EBS and IBS sequences, retrohoming occurs by TPRT. The intron RNA cleaves the sense strand at the exon junctions, and integrates into the target site. At the same time, the IEP cleaves the antisense strand through its EN activity (for the Ll.ltrB, this cleavage occurs at position +9). The 3′ end of the antisense strand is used by the RT domain of the IEP as the primer site for reverse transcription of the inserted intron RNA. The intron cDNA is then integrated into the host target site by homologous recombination-independent repair mechanisms. Some mobile group II introns, such as the *S. meliloti* RmInt1 intron (IIB3/class D), have an IEP with no En domain. The C-terminal maturase region (C-tail) of this intron is responsible for its maturase and DNA-insertion functions (Molina-Sánchez et al., 2010). The RmInt1 IEP has RT activity, and its RNP can mediate reverse splicing into target sites at double- or single-stranded DNA substrates but cannot achieve site-specific second-strand cleavage (Muñoz-Adelantado et al., 2003). An alternative to the TPRT retrohoming pathway is therefore required. RmInt1 retrohomes very efficiently (Martínez-Abarca et al., 2000; Martínez-Abarca and Toro, 2000a,b; Nisa-Martínez et al., 2007), and its mobility is dependent on the intron RNA and the IEP, recognizing a target site extending 20 nt into the 5′ exon and 5 nt into the 3′ exon (Jiménez-Zurdo et al., 2003). The preferred retrohoming pathway of RmInt1 involves the reverse splicing of the intron RNA into single-stranded DNA at replication forks, with a bias toward the template for lagging strand synthesis (Martínez-Abarca et al., 2004), probably through the use of the RNA primers synthesized by the primase or the Okazaki fragments to prime reverse transcription (**Figure 1B**, down, right side).

## RmInt1 and Closely Related Introns in *S. meliloti* Control the Spread of Potentially Harmful Mobile Genetic Elements

The *S. meliloti* genome harbors many different mobile genetic elements, including group II introns from classes C, D, E, and G (Toro et al., 2002, 2003; Candales et al., 2012; Toro and Martínez-Abarca, 2013). RmInt1 is widespread in *S. meliloti* (Muñoz et al., 2001) and was first described in the GR4 strain (Martínez-Abarca et al., 1998), which contains 10 copies of RmInt1 (Martínez-Abarca et al., 2013) distributed between the different replicons [four copies on the chromosome, five copies on pRmeGR4c (pSymA), and one copy on the accessory plasmid pRmeGR4b]. This bacterial strain also harbors a closely related mobile intron called RmInt2 (Martínez-Rodríguez et al., 2014). RmInt2 also belongs to class D, it has a nucleotide sequence 72% identical to that of RmInt1, and it is present in seven copies (four copies on pSymA and three on pSymB). Other group II introns are present as single copies in this bacterial genome: a class C intron (pSymB IEP: WP_015243078.1), a class G intron (chromosome IEP: WP_015241266.1), and a disrupted class E intron (S.me I4) (Toro et al., 2002) located on the accessory plasmid pRmeGR4b. It is thought that 90% of *S. meliloti* isolates harbor RmInt1 intron, with copy numbers differing between *S. meliloti* strains. Full-length close relatives of RmInt1 (85–99% nucleotide identity) are also present in *S. medicae*, *E. adhaerens* and *S. terangae* (closest relatives of *S. meliloti*), whereas closely related fragmented introns have been identified in these bacterial species and in other *Sinorhizobium* and *Rhizobium* species (Fernández-López et al., 2005). These RmInt1-like elements were likely acquired by vertical inheritance from a common ancestor and through independent lateral transfer events.

**FIGURE 2:**
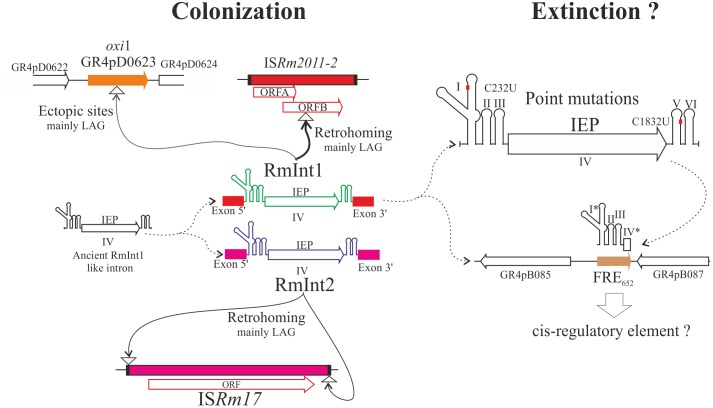
Different scenarios of intron spread within *S. meliloti*. Colonization phase: consisting mostly of the movement of functional introns (RmInt1 and RmInt2) to their respective canonical targets (IS*Rm2011-2* and IS*Rm17*) via retrohoming or to non-canonical targets (ectopic sites) into targets mainly located in the DNA strand used as template for the lagging strand (mainly LAG) present in different replicons. Extinction phase: once several point mutations have occurred (exemplified by two particular changes to the ribozyme and intron; Molina-Sánchez and Toro, 2015), the element is no longer able to move, and may also degenerate through the loss of most of the IEP-encoding sequence (exemplified by the FRE_652_ element; Toro et al., 2014b). The maintenance of FRE_652_ in the population suggests that it may act as a *cis* element in this particular genomic context. Arrows indicate the direction of movement of functional introns. The dashed curved arrows show the inferred ancestor-descendant relationships. The asterisk indicates that the domains (I and IV) are truncated.

The natural target site of RmInt1 lies within IS*Rm2011-2* (IS*Rm11*) an insertion sequence from the IS630 family (**Figure 2**; Selbitschka et al., 1995), and this intron is also found in other closely related insertion elements (Toro et al., 2003; Biondi et al., 2011). IS*Rm2011-2* is present virtually in all *S. meliloti* strains, usually at high copy numbers (4–13; Biondi et al., 2011). In its natural host, RmInt1 has a very low splicing ability *in vivo*; insertion of the intron into IS*Rm2011-2* therefore blocks its spread (Chillón et al., 2011). Group II introns are highly flexible in terms of their ability to colonize the *S. meliloti* genome (Nisa-Martínez et al., 2007). For example, RmInt2 has a lower retrohoming efficiency than RmInt1, and a greater probability of survival and spread in the genome through the relaxation of target-site specificity using the left and right inverted repeats of IS*Rm17* as DNA targets (**Figure 2**), thereby ensuring that at least one of its targets is on the template of the lagging strand during DNA replication (Martínez-Rodríguez et al., 2014). The spread, gain, and losses of these IS elements not carrying adaptive traits likely impose costs on their hosts in the short term, particularly when their copy number increases, generating a source of genetic instability and a burden for the cell replication machinery (Rankin et al., 2011). The mobile introns that use these elements as a target not only help the host cell to reduce such fitness costs by limiting their ability to spread within the genome, but also contribute to their coexistence with the host genome, allowing time for selective forces to exert their effects on both the mobile elements and the host genome. Thus, group II introns in bacteria, and RmInt1 in particular, may be considered key elements in the short- and long-term evolution of the *S. meliloti* genome.

## Ectopic Transposition of RmInt1 as a Source of Early Genomic Variation in *S. meliloti*

As indicated above, group II introns can insert into intronless alleles at intron-specific locations (homing), and at novel (ectopic) sites, albeit at low frequency, and this has been identified as an important mechanism of intron spread in bacterial populations (Muñoz et al., 2001). Initial studies on the GR4-type *S. meliloti* population showed that RmInt1 was able to invade the ectopic *oxi1* site. The intron is able to recognize this target site because this gene carries IBS sequences similar to those of IS*Rm2011-2*. Following the sequencing of the genome of GR4 (Martínez-Abarca et al., 2013), *oxi1* was identified in pRmeGR4d (pSymB) as a gene encoding an oxidoreductase (WP_010975806.1), resulting from an ectopic transposition event that has occurred independently several times in the natural population (Toro et al., 2016). In the GR4-type population analyzed, 4.1% of the isolates displayed such intron insertions, but this variant is fixed in 95% of EM2-type isolates, a different population obtained from nodules also occupied by GR4-type strains, suggesting that it may be subject to selective pressure (Muñoz et al., 2001). Moreover, whole-genome sequencing in GR4-type isolates identified another ectopic site, an intron insertion in a gene encoding a NAD(P)-dependent oxidoreductase (WP_010969452.1), corresponding to an event that probably occurred about 1700 years ago (Toro et al., 2016, 2017). The biological significance of the targeting of these genes by RmInt1 in *S. meliloti* is unknown. The characterization of these loci, together with genome-wide sequence analysis of the EM2-type population, will therefore provide insight into the importance of the ectopic transposition of group II introns for early genome evolution in natural bacterial populations.

## The Dynamics of RmInt1 in *S. meliloti*

The *S. meliloti* strains lacking RmInt1, such as RMO17 (Toro et al., 2014a), have been shown to remain suitable for intron colonization. The arrival of the intron is followed by its spread through high-frequency retrohoming (using an RNA intermediate) with a strand bias related to the DNA replication (Nisa-Martínez et al., 2007). Like other genetic mobile elements, group II introns appear to follow a gain-loss cycle that would account for the frequency and copy number of RmInt1 in *S. meliloti* species (Molina-Sánchez and Toro, 2015). It has been suggested that bacteria with highly colonized genomes by a particular intron are removed from the population by purifying selection in genome-wide selective sweeps (Leclercq and Cordaux, 2012). This elimination probably occurs due to the genomic instability resulting from possible recombination between intron copies, leading to the deletion of parts of the genome. For RmInt1, gradual eradication (**Figure 2**) begins with specific mutations of the intron ribozyme RNA and the IEP, inactivating intron splicing and retrohoming (Molina-Sánchez and Toro, 2015). This inactivation is followed by further fragmentation, with the loss of the intron 3′-end, including the IEP (Dai and Zimmerly, 2002; Fernández-López et al., 2005), which seems to be a general process underlying the loss of function of bacterial group II introns. This gradual process of inactivation may still result in a transitory active mobile element rescued by either the intron RNA or IEPs from other active copies present or arriving to the same cell. Nonetheless, this process is unlikely to remove intron sequences completely (**Figure 2**). Instead, some intron fragments are likely to remain buried within the genome. For example, a 652 nt RmInt1-like fragment known as FRE_652_, which is 88.9% identical to RmInt1 and 89.7% identical to the *S. medicae* intron Sr.md.I1, has persisted over long periods of evolutionary time in the genomes of *S. meliloti* and *S. medicae* (Toro et al., 2014b). This intron fragment covers ribozyme DI–DIII and includes part of DIV, truncated at a position corresponding to position 653 of RmInt1. FRE_652_ is located on the accessory plasmid pRmeGR4b of strain GR4, whereas it is carried by pSymA and orthologous plasmids in other *S. meliloti* and *S. medicae* strains, respectively. FRE_652_ is located close to a predicted helix–turn–helix transcriptional regulator and a diguanylate cyclase/phosphodiesterase (DGC/PDEA) gene, and is followed by a pectate lyase and a carbonate dehydratase. All these genes are absent from *S. meliloti* strains 1021 and Rm41. It therefore seems likely that FRE_652_ and the surrounding loci are involved in recombination events within the various replicons of the multipartite genome of these bacterial species and that this element may even spread within populations, through recombination, without causing genome-wide selective sweeps. The significance and impact of fragmented introns within a particular genome remains uncertain, but we suggest that FRE_652_ and other intron fragments have persisted and are continuing to evolve in the genome perhaps by contributing to the environmental adaptation and symbiotic capacity of these rhizobial species, perhaps as *cis*-regulatory elements (**Figure 2**).

## Conclusion

The presence of active mobile introns, such as RmInt1 and close relatives, in the *S. meliloti* genome constitutes an interesting model for exploring the dynamics and possible influence of these retroelements in bacterial genome evolution. We suggest that the persistence of these self-splicing catalytic RNAs in bacteria, such as RmInt1 in *S. meliloti*, results from their possible involvement in short- and long-term mechanisms underlying genome evolution. We therefore consider these apparently “parasitic” genetic elements as a source of direct and indirect genetic variation contributing to genome evolution and ecological differentiation in natural bacterial populations.

## Author Contributions

NT prepared the manuscript. MM-S and FG-R prepared the **Figure 1**, and FM-A prepared the **Figure 2**. All authors critically reviewed the manuscript.

## Conflict of Interest Statement

The authors declare that the research was conducted in the absence of any commercial or financial relationships that could be construed as a potential conflict of interest.
